# Percutaneous transthoracic catheter drainage prior to surgery in treating neonates with congenital macrocystic lung malformation presenting with respiratory distress

**DOI:** 10.3389/fped.2023.1268028

**Published:** 2023-11-23

**Authors:** Taozhen He, Xiaoyan Sun, Dengke Luo, Shiyi Dai, Miao Yuan, Gang Yang, Kaisheng Cheng, Chang Xu

**Affiliations:** ^1^ Department of Pediatric Surgery, West China Hospital of Sichuan University, Chengdu, China; ^2^ Health Management Centre, West China Hospital of Sichuan University, Chengdu, China

**Keywords:** thoracic drainage, respiratory distress syndrome, congenital macrocystic lung malformation, cystic adenomatoid lung malformation, neonates

## Abstract

**Backgound:**

It is rarely seen that neonates with congenital macrocystic lung malformation (CMLM) presenting with respiratory distress require emergency intervention. No consensus has been achieved concerning the best policy facing such condition. This study aims to evaluate the efficacy and safety of our strategies in treating neonates with CMLM presenting with respiratory distress.

**Methods:**

We retrospectively reviewed the data of six neonates with CMLM presenting with respiratory distress from April 2020 to October 2022 for whom drainage-prior-to-surgery strategy were adopted and favorable outcomes were obtained. The relevant data was reviewed and analyzed.

**Results:**

All the patients were prenatally diagnosed with congenital lung malformation and postnatally as congenital macrocystic lung malformation via CT scan. Each neonate accepted percutaneous thoracic catheter drainage prior to surgery. The first and fifth neonates with macrocystic lung mass experienced prompt open lobectomy and delayed thoracoscopic surgery due to failure of air drainage, respectively. The other four patients obtained good drainage of the large air-filled cyst, thus gaining the opportunity for elective thoracoscopic surgery within median 45 days.

**Conclusions:**

For neonates with macrocystic lung malformation presenting with respiratory distress due to mediastinal compression, percutaneous thoracic catheter drainage is worth a shot for elective thoracoscopic surgery due to its feasibility and safety.

## 1. Introduction

Congenital lung malformations (CLM) are a group of anomalies that are characteristic of pulmonary abnormalities, including congenital pulmonary airway malformation (CPAM) pulmonary sequestration, bronchogenic cyst and lobar emphysema, in which CPAM accounts for the largest proportion. The majority of children diagnosed with CPAM are asymptomatic at birth, and elective pulmonary resection beyond the neonatal period is recommended and associated with favorable outcomes (1–5). In addition, there is a small group of patients with macrocystic CPAM who have early symptoms, manifesting as significant respiratory distress within the neonatal period secondary to mass effects on the heart and lungs (6–8). These neonates are supposed to undergo prompt interventions after birth. Emergency surgery was the mainstay for rescuing them despite a higher anesthetic risk (9–11). Besides, od-transthoracic puncture and drainage of the large cyst followed by elective surgery was once reported (12–14), although rare, in the literature and resulted in excellent outcomes. The latter seems to be more appropriate in stabilizing the neonates using a simple maneuver in emergency. To verify this, we presented our experiences on macrocystic CPAM neonates with respiratory distress and evaluated their safety and efficacy and attempted to delineate an algorithm based on our experiences and the literature.

## 2. Materials and methods

### 2.1. Study design and participants

A retrospective chart review was performed to evaluate the congenital macrocystic lung malformation (CMLM) neonates presenting with respiratory distress in the Department of Pediatric Surgery, West China Hospital, Sichuan University, from April 2020 to October 2022. The study was approved by the Research Ethics Board of Sichuan University West China Hospital. All subjects participating in the study gave written informed consent. We included prenatally diagnosed CMLM neonates presenting with progressive respiratory distress after birth due to mediastinal shift and lung compression caused by large space-occupying lesions. We excluded those with concomitant diseases that can also cause respiratory distress, such as pneumonia, congenital cardiac anomalies, and upper respiratory tract obstruction. Medical and operative records were retrieved for data analysis. Patient data including sex, weight, Apgar score, lesion size, CPAM volume ratio(CVR), timing of respiratory distress onset, age at PTCD, age at surgery, surgery type, time to discharge after surgery and complications were extracted and analyzed. Postoperative complications were defined as pneumothorax, tube dislodgement, failure to drain, broncho-pleuric fistula, persistent air leak (≥7 days), major bleeding, and subcutaneous emphysema.

### 2.2. Techniques

#### 2.2.1. Seldinger maneuver

After lidocaine infiltration a pigtail tube was used through Seldinger manoeuvre, based on preoperative CT scan images. Success of the PTCD maneuver was defined as continuous drainage of trapping air from the lung cyst via the tube, and complete relief of respiratory distress was obtained (SpO2 increase to above 95% without pure O2 uptake). Failure to drain included (1) tube was placed in pleural cavity rather than in large cyst and (2) Tube was placed in the cyst not connecting to the bronchi. At this time, another attempt was reasonable. Thoracoscopic lobectomy through the hilar approach was applied in elective surgery, and thoracotomy in emergency surgery. The details of lobectomy have been described in our previous study (15)

## 3. Results

**Figure 1:**
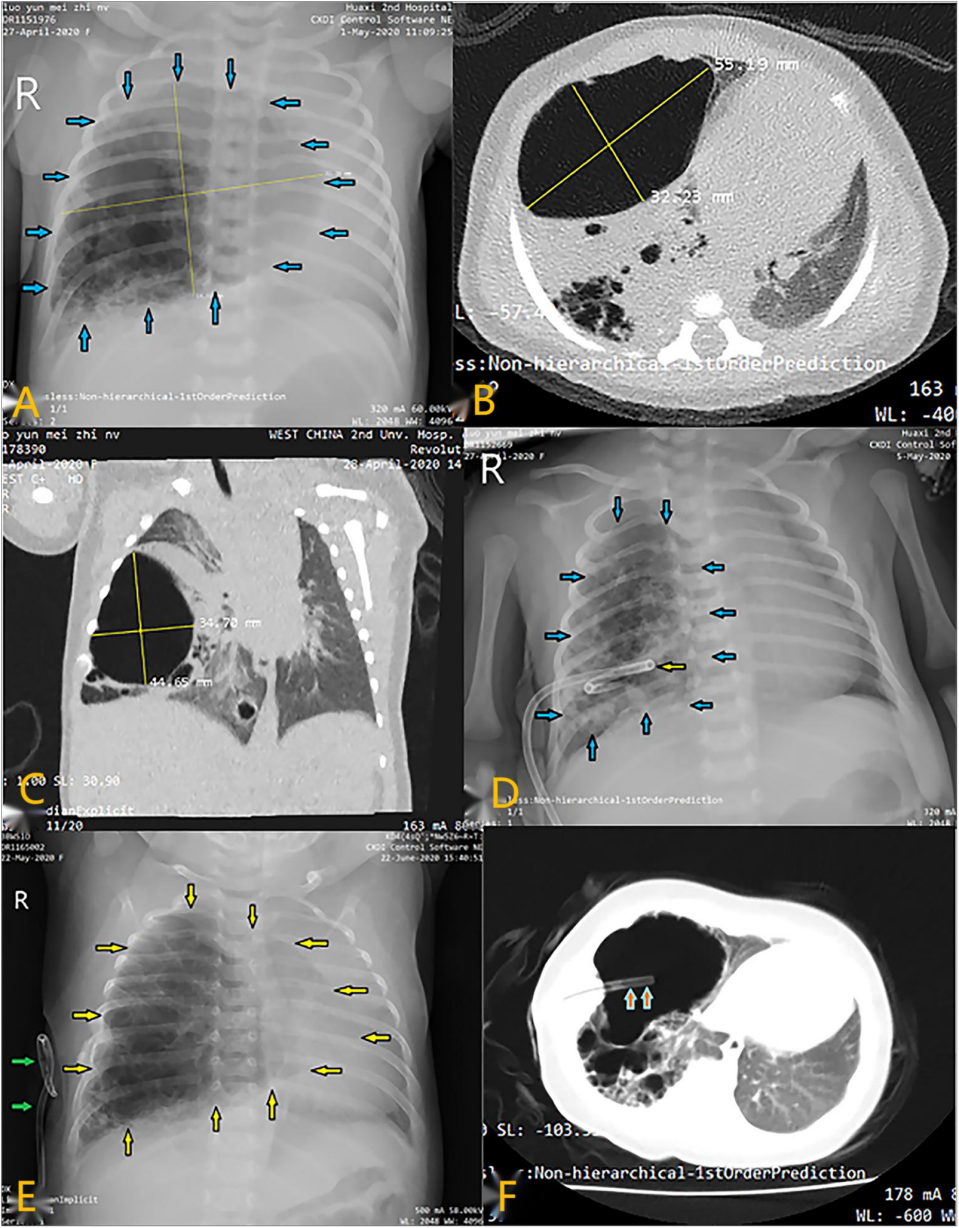
Radiological imaging of PTCD maneuvre in case 2. (**A**) Preoperative CT scan showed a large cystic lung lesion in the left lower lobe; (**A–C**) X-ray image showed a large cystic lesion (blue arrows) in the right thoracic cavity; CT scan indicated that the cystic lesion was so large that mediastinal displacement occurred; (**D**) Chest film demonstrated that a pigtail catheter (yellow arrow) was successfully placed in the lesional cyst; (**E**) Chest X-ray showed that the lesional cyst (yellow arrow) was restored to its original size when the catheter (green arrow) dislodged out of it; (**F**) CT scan indicated suction catheter (orange arrow) was successfully placed into the large cyst along the existing fistula in the thoracic wall.

**Figure 2:**
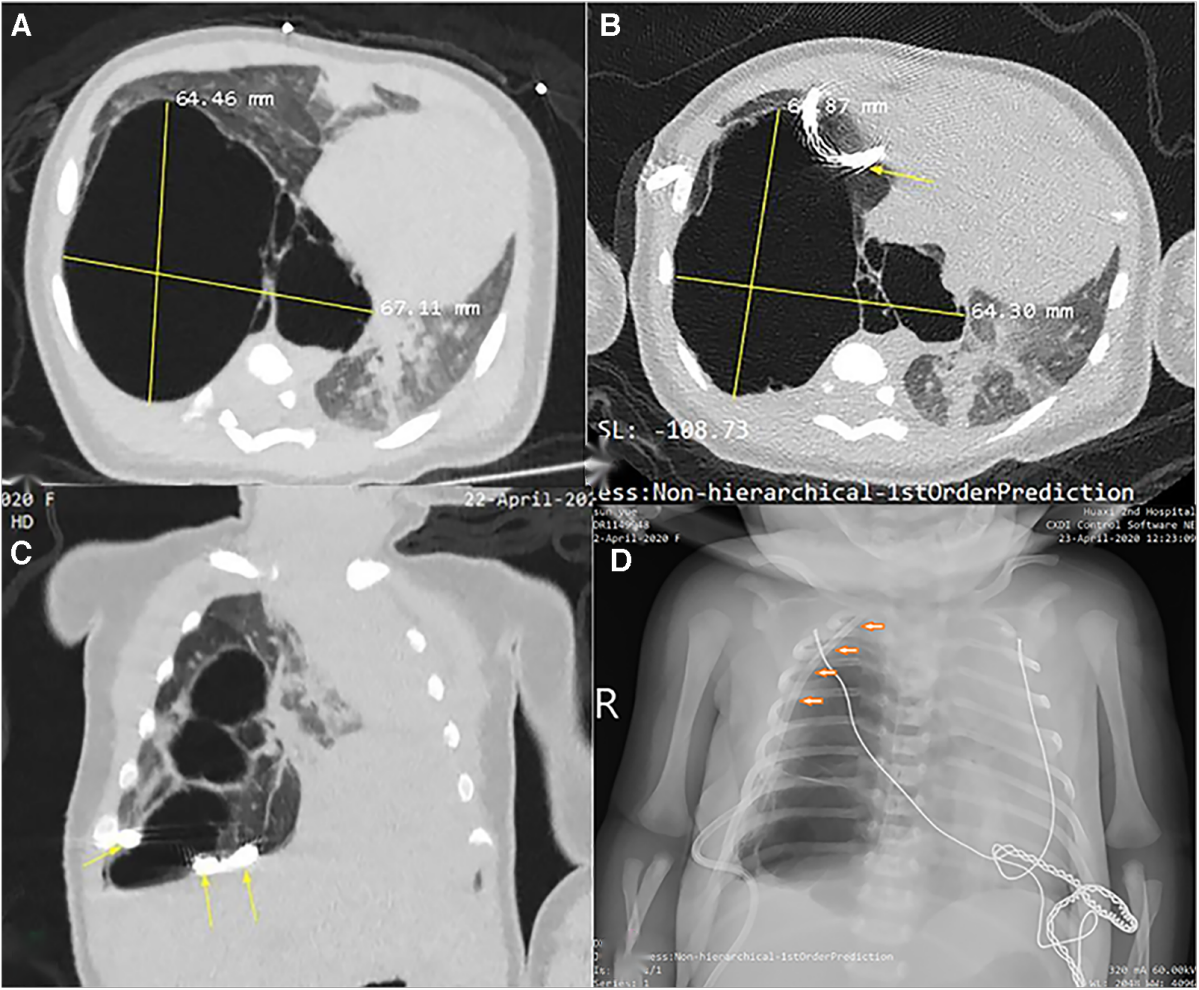
Radiological imaging of PTCD maneuvre in case 1. (**A**) Axial view of CT scan prior to puncture indicated a huge cystic lung lesion with septa in the right thoracic cavity; (**B**) and (**C**) CT scan demonstrated the pigtail catheter (yellow arrow) was placed (first attempt) outside of the large cyst; (**D**) Chest X-ray film displayed the tube was positioned into the thoracic cavity rather than the large cyst by second attempt.

**Figure 3:**
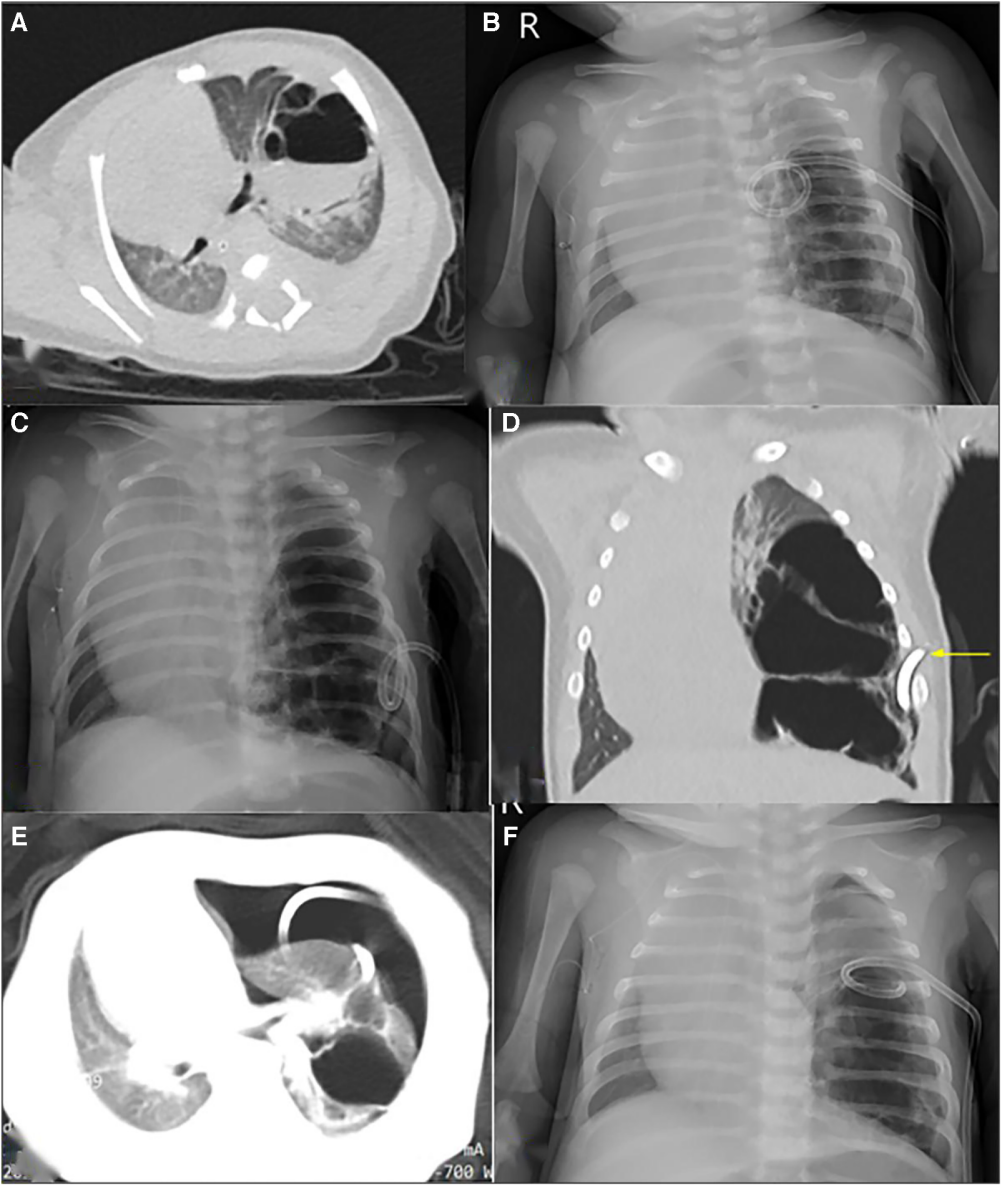
Radiological imaging of PTCD maneuvre in case 5. (**A**) Preoperative CT scan showed a large cystic lung lesion in the left lower lobe, during which an liquid-air level was noted; (**B**) and (**C**) The first and second post-PTCD radiological image both displayed little relief of mass effect, respectively; (**D**) CT scan indicated the lung lesion was multicystic and could not be completely decompressed by the pigtail catheter (yellow arrow); (**E**) CT scan shown pneumothorax occurred secondary to the 2nd PTCD; F: Chest X-ray indicated the air in the thoracic cavity was drained via catheter adjustment.

A total of six patients were included. All of them were prenatally diagnosed with CLM and classified as MCLM after birth. They all undergo PTCD before surgery. All of the neonates presented respiratory distress (ranging mild to severe) immediately after birth. Neonates (Case 1 and 5) seemed to respond well with respiratory support in the first one to two weeks and underwent first PTCD at d 19 and d 8, respectively. The remaining patients (case 2, 3, 4, 6) presented tachypnea (60 times per min above) and the SPO_2_ could not be maintained above 95% by oxygen mask ventilation. Therefore, they promptly underwent PTCD on the first day after birth and obtained good drainage of the large air-trapping cyst (Figure 1), thus gaining the opportunity for elective thoracoscopic surgery in median 45 days (range from 35 to 60 days), during which lobectomy were smoothly performed. In Case 1, the neonate experienced prompt open lobectomy due to failure to place chest tube in the air-trapping cyst after two PTCD attempts (Figure 2). This patient obtained a rapid recovery with extubation on the 2nd day after surgery and was discharged home on the 9th postoperative day. In the 5th case, PTCD was performed twice with interim relief of respiratory distress (Figures 3B–D). Pneumothorax occurred after the second Seldinger maneuver, which indicated air from a ruptured cyst entered the pleural cavity (Figure 3E), thetube was removed and another one was inserted to drain the free air and partial lung expansion was obtained (Figure 3F). However, the mass effect of the macrocystic lung lesions did not seem to be alleviated. So emergency thoracoscopic lobectomy was performed on the 36th day after birth and favourable outcome was obtained.

Catheter dislodgement occurred 3 weeks after PTCD maneuver in case 2 and 3 days after PTCD in case 6, respectively. Replacement of chest tube were successfully performed and maintained for a few weeks. They all recovered well, and the relevant data are shown in Table 1. All patients were diagnosed with macrocystic CPAM according to the pathological findings. The follow-up (median 18 months) was uneventful. Literature concerning macrocystic CPAM neonates who underwent emergency interventions because of mass effect was reviewed and listed in Table 2.

**Table 1:** Characteristics of CMLM neonates undergoing emergency PTCD and/or surgery in current study.

Case No	Year	Birth weight (g)	GA (wk)	Apgar score (1 min, 5 min)	Lesion size (cm)	CVR	TRDO	Age at PTCD (d)	Age at surgery	Surgery type	TDAS (d)	Complication	FU (mo)	Death
1	2020	3,550	38	9, 10	4.8	1.7	IAB	19/22	23	OL	9	Failure to drain	16	No
2	2020	3,160	36	9, 10	6.5	4.5	IAB	1	60	TL	8	Tube dislodgement	18	No
3	2021	3,325	38	8.9	5.5	3.7	IAB	1	35	TL	7	None	20	No
4	2021	3,150	38	9.10	6.0	2.5	IAB	1	48	TL	8	None	25	No
5	2022	4,130	39	10.10	5.3	2.1	IAB	8/18	36	TL	20	Failure to drain, Pneumothorax	6	No
6	2022	3,250	39	10.10	5.4	2.4	IAB	5	42	TL	4	Tube dislodgement, Pneumothorax	3	No

CLM, congenital lung mass; GA, gestational age; TDAS, time to discharge after surgery; PTCD, percutaneous transthoracic catheter drainage; CVR, CPAM volume ratio; TRDO, timing of respiratory distress onset; IAB, immediately after birth; OL, open lobectomy; TL, thoracoscopic lobectomy; LUL, left lower lobe; RLL, right lower lobe; RUL, right upper lobe; LLL, left lower lobe; FU, follow up.

**Table 2:** Characteristics of CMLM neonates undergoing emergency intervention due to mass effect in the literature.

Case no	Year	Author	Gestational age (wk)	Birth weight (g)	Apgar score (1 min, 5 min)	Lesion size (cm)	Age at Surgery (d)	Surgery type	TDAS (d)	Complication	Death
1	1994	David	37	3,525	6, 8	4.0	6	OL	7	U	No
2	2002	Allegaert	37	4,040	9, 9	U	1	OL	12	U	No
3	2009	Lecomte^a^	Mean 40	U	> 7	U	1/14	OL	None	Air leak (*n* = 1), Infection (*n* = 1)	Yes^f^
4	2014	Manuela	37/35.5	3,900/2,600	U	U	7	OPLR	U	Sepsis	Yes^g^
5	2016	Chong	31	1,590	7, 9	U	24	OL	21	None	No
6	2017	Seong^b^	37.4 (34.6–39.1)	2,710 (2,304-3,000)	6 (4.5–8), 8 (7–9)	5.6 (4.2–6.9)	4 (1–12)	OL	13 (1–26.5)	U	No
7	2018	Makhijani	U	U	U	U	19	OL	12	pneumothorax	No
8	2019	Ito^c^	U	2,850 (2,500–3,225)	U	U	5.5 (3.25–7.25)	OS^e^	15.5 (14.75–16)	Cystic remnant (*n* = 1)	No
9	2019	Disu	U	3,100	U	U	21	OL	7	None	No
10	2020	Huang	39	2,499	U	5.7	4	TL	16	None	No
11	2022	He^d^	38 (38–38.75)	3,287.5 (3,182.5 −3,493.75)	9 (9,9.75), 10 (10,10)	5.45 (5.325,5.875)	39.0 (35.25–46.5)	OL(*n* = 1), TL(*n *= 5)	7.5 (5.5–8.0)	See Table 1	No

CCLM, congenital cystic lung mass; TDAS, time to discharge after surgery; PTCD, percutaneous transthoracic catheter drainage; CVR, CPAM volume ratio; OL, open lobectomy; TL, thoracoscopic lobectomy; LUL, left lower lobe; RLL, right lower lobe; RUL, right upper lobe; LLL, left lower lobe; R, right; L, left; OPLR, open partial lung resection; U, unmentioned.

^a^ Two cases included.

^b^ Seven case series.

^c^ Four case included.

^d^ The current study.

^e^ Open surgery including lobectomy, segmentectomy, wedge resection and fractionated lung resection.

^f^ Died of respiratory failure caused by infection on day 20 and by air leak on day 15, respectively.

^g^ Died of sepsis.

## 4. Discussions

In fact, emergency thoracotomy worked as the most frequent intervention (4, 6, 10, 11, 13, 16–20) with a relatively higher anesthetic risk, which, to some extent, may lead to an unfavorable outcome. Lecomte B. et al. (10) reported 10 cases of CPAM; two neonates with respiratory distress due to mediastinal shift and polyhydramnios underwent emergency surgeries and died of respiratory failure caused by infection on day 20 and by air leak on day 15, respectively. Manuela and colleagues (19) reported one neonate with disseminated bronchial crackles and productive cough who received two emergency surgeries and died of sepsis. Elective resection in asymptomatic infants is advised to reduce the risk of postoperative complications (2). Zheng et al. (8) retrospectively reviewed 24 medical records of neonates who underwent surgery for symptomatic CPAM from 2010 to 2020 and yielded excellent clinical outcomes after symptoms of respiratory distress had adequately subsided. Moreover, elective surgery allows thoracoscopic resection, which can provide a shorter hospital stay, decreased postoperative pain and better cosmesis (1, 3). In contrast, fifteen of eighteen neonates (83.3%) from the obtained literature (including the current study) underwent emergency thoracotomy rather than the thoracoscopic approach, mainly due to the narrow thoracic space. The thoracic cavity of the neonates would enlarge significantly within couple of weeks, thus facilitating thoracoscopic surgery. In the literature we reviewed, 17 of 18 (94.4%) newborns underwent lobectomy instead of parenchyma-preserving surgery despite the great compensatory potential of the residual lung. Unfortunately, there was only one case (4) in which segmentectomy was performed, thus complicating cystic remnant. Normally, it is a great challenge for any surgeon to perform parenchyma-sparing surgery on a neonate in an emergency setting. Although no lung-sparing surgery was performed in this study, PTCD was regarded as our preference for these neonates due to lower anesthetic risk and the latent benefits of elective surgery (thoracoscopic approach and lung-preserving possibility). As a result, PTCD and surgery was considered to be a better alternative to emergency thoracotomy (2), which was supported by this case series.

In the literature, PTCD was performed on a clinical basis with CT image navigation (13, 14) or under fluoroscopic guidance (20). We considered it adequate for a skilled surgeon to perform this maneuver based on CT images We looked back to case 1, in which the PTCD maneuver should be deemed as failure, for the catheter seemed to be placed outside of the cyst (probably inside the pleural cavity, see Figures 2B,C). Therefore, to raise the success rate of PTCD, radiological confirmation of tube placement is mandatory.

Indeed, PTCD of a lung cyst is different from thoracocentesis to some degree. Most likely, the puncture needle can easily penetrate the cyst wall. However, the pigtail tube could fail to advance along the guidewire because the cystic wall is, unlike the thoracic wall, elastic and unfixed while it advances. Retrospectively, the failure of PTCD in case 1 could be attributed to this explanation. The puncture site, angle and adequate depth of advancement are of great importance. To avoid complications pertaining to lung injury, the puncture point should be a proper surface site to which the target cyst wall is mostly adjacent with the least lung parenchyma interposition between the inner thoracic wall and the cyst. The needle should be perpendicular to the cyst surface. Oh SH et al. (20) reported complications of PTCD in seven cases, including pneumothorax (*n* = 2), catheter displacement (*n* = 1) and failure to drain (*n* = 1). They reported pneumothorax occurred due to use of a non-pigtail catheter (chest tube or angiocatheter). Similar to the complications of PTCD reported above, Pneumothorax (*n* = 1), catheter displacement (*n* = 2), failure to drain (*n* = 2) and broncho-pleuric fistula (*n* = 0) occurred in PTCD using pigtail catheter in the current study. We noted that failing to drain both occurred in the two cases (case 1 and case 5), the former case had faster postoperative recovery than the latter one (time to discharge home after surgery, 9 days vs. 17 days) even though open surgery was performed in case 1. Maybe the duration of respiratory distress prior to surgery (23 days vs. 36 days) could explain. We assume that the shorter the duration of respiratory distress was, the less impairment to the remaining lung was, the faster the neonate recovered, which needs to be verified in further study.

Maintaining the chest tube after discharge needs careful parental care to avoid a significant number of severe complications. In this study, 2 babies experienced tube dislodgement, thus causing recurrence of respiratory distress. Fortunately, the respiratory symptom relieved again by reinserting another tube along the external opening of the fistula at the thoracic wall, which lasted couple of weeks prior to surgery. To a certain degree, we overestimated the parents' nursing abilities in securing the chest tube at home for a long time (4–8 weeks).

Although Oh SH, et al. (20) reported that the mean time between PCTD and surgery was 4 days only, they all underwent open lobectomies, which meant greater trauma and potential long-term complication such as chest wall deformity and scoliosis. Narrow thoracic space is an obstacle of thoracoscopic surgery, therefore, we expect the babies to gain more weight, greater thoracic cavity and more mature lung (better tolerating the anesthetic risk), thus facilitating the thoracoscopic surgery. Just as one coin has two sides, although the babies staying at home with chest tube in the body take more risk (hemothorax or dislodgement of chest tube) to a certain extent, the time to discharge home in the current study is shorter (median 8 days vs. 13 days). Beside, the parents strongly requested for thoracoscopic surgery for their babies after full consideration. As such, to decrease the take-tube-home risk, several conditions should be met. Firstly, the parents' compliance should be good enough to master some nursing techniques. Secondly, the patients' home are not far away from the clinics or hospitals where professional medical care are available in emergency. Thirdly, the time of staying home with tube-in-the-body should not be within 1 month depending on the parents' compliance.

**Figure 4:**
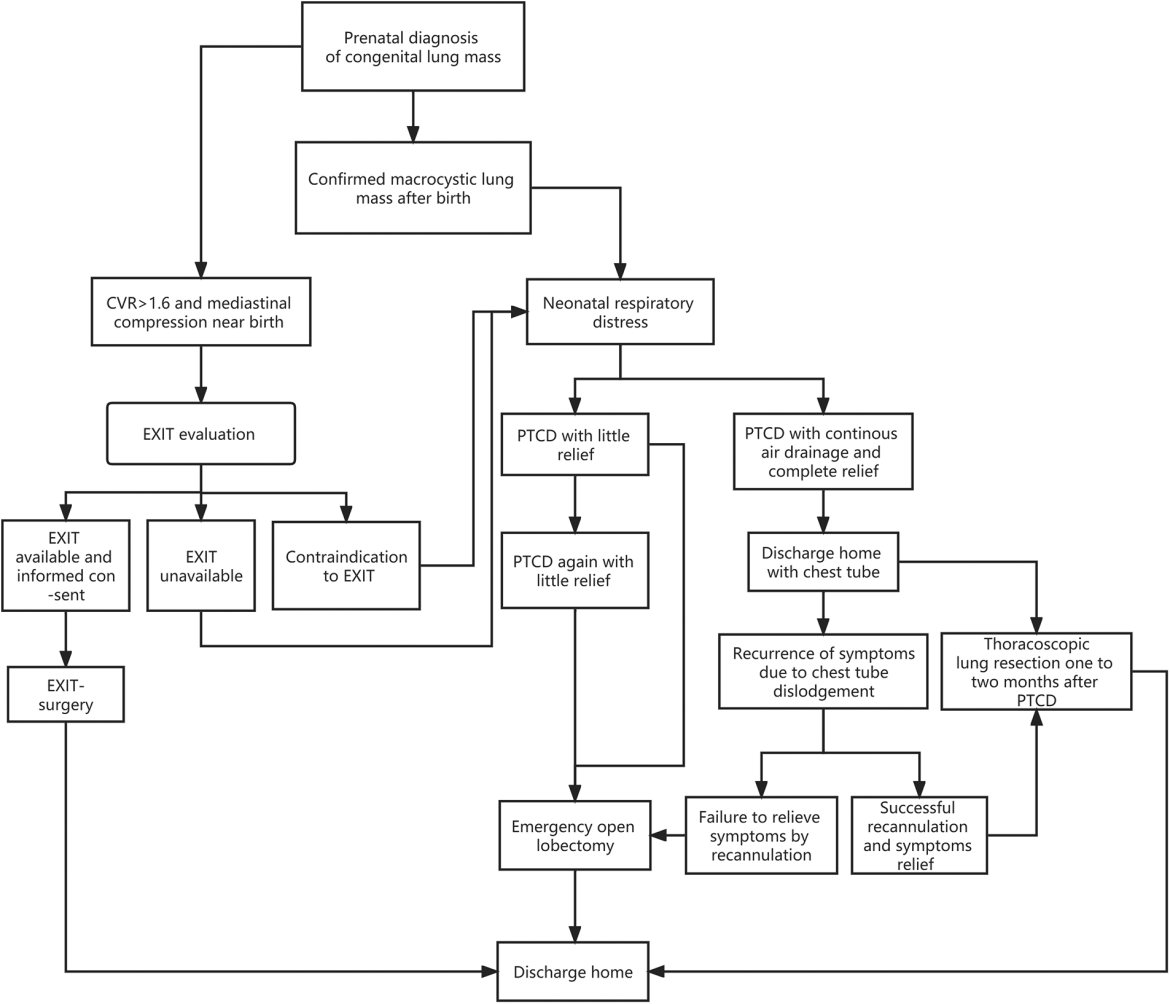
Algorithm of a prenatally diagnosed congenital lung macrocystic malformation that may manifest with respiratory distress after birth.

It is worth mentioning that Cass DL and colleagues (21) reported that nine prenatally diagnosed CLM newborns comprised of five cystic masses (all macrocystic CPAM) and four solid masses with persistent mediastinal compression treated with ex-utero intrapartum treatment (EXIT) were well discharged at a median of 10 d postoperatively. Similar to the strategy we adopted, EXIT is, to some degree, equivalent to an elective (prophylactic) surgery with full preparation for neonatal delivery and establishment of the respiratory tract prior to dissection of the umbilical cord, thus preventing neonatal respiratory distress. However, conducting an EXIT-to-resection requires multidisciplinary cooperation, which is generally integrated into a tertiary medical center with advanced equipment and specialists, which is not suitable for primary-level medical institutions. In other words, the PTCD procedure could be deemed a first-aid intervention in basic medical units with a higher benefit-risk ratio. Based on our experiences and the literature, an algorithm of neonatal CMLM with respiratory distress was delineated (Figure 4), which could be referenced by others.

Several caveats regarding this study should be acknowledged. Firstly, this was a retrospective, single institution series with a relatively short follow-up period (median 18 months). Secondly, the study population was rather small due to the low morbidity rate. Thirdly, a lack of control will no doubt degrade the evidence level of this study. Last but not least, since the majority of neonatal units' standard policy is to remove the chest tube before discharge, the clinical algorithm mentioned in this study can not be referenced in many low and middle income countries. Despite these limitations, this was the first paper that exclusively delineated an algorithm of prenatally diagnosed CMLM that may cause respiratory distress after birth. Case control study with larger sample size is anticipated in the future.

## 5. Conclusions

For neonates with macrocystic lung malformation presenting with respiratory distress due to mediastinal compression, percutaneous thoracic catheter drainage is worth a shot for elective thoracoscopic surgery in selected cases, which is deemed as a safer alternative to direct emergency thoracotomy.

## Data Availability

The raw data supporting the conclusions of this article will be made available by the authors, without undue reservation.
